# Mobilität, Autofahren und funktionale Kompetenz bei älteren Menschen – Ausgewählte Ergebnisse einer Längsschnittstudie zur Alterung urbaner Kohorten (LUCAS)

**DOI:** 10.1007/s00103-024-03921-6

**Published:** 2024-07-17

**Authors:** Wolfgang von Renteln-Kruse, Klaus Püschel

**Affiliations:** 1 Albertinen-Haus , Medizinisch-Geriatrische Klinik, Hamburg, Deutschland Süntelstr. 11 a,; 2 Institut für Rechtsmedizin, Universitätsklinikum Hamburg-Eppendorf (UKE), Butenfeld 34, 22529 Hamburg, Deutschland

**Keywords:** Alter, Mobilität, Funktionale Kompetenz, Autofahren, Fahreignung, Age, Mobility, Functional competence, Car driving, Car driving ability

## Abstract

Mobilität ist auch im höheren Alter maßgeblich für die selbstständige Lebensführung. Ältere Menschen beginnen bei geringerer körperlicher Leistungsfähigkeit (Gebrechlichkeit, engl. Frailty) ihren persönlichen Aktionsradius auf das nähere Wohnumfeld und schließlich die unmittelbare Häuslichkeit zu begrenzen. Erkrankungen des Bewegungsapparates, neurologische, psychische, kognitive, Sinnes- oder Kreislaufstörungen können die funktionale Kompetenz (Fähigkeit, unabhängig zu leben) einschränken.

In einer Längsschnittstudie zur Alterung urbaner Kohorten (LUCAS), aus der in diesem Artikel ausgewählte Ergebnisse berichtet werden, wurden die Teilnehmenden mittels des LUCAS-Funktions-Index unterschiedlichen Funktionsklassen zugeordnet (*Robust, postRobust, preFrail* und *Frail*). Die Ergebnisse zeigen, dass zunehmende Verluste funktionaler Kompetenz mit abnehmender Mobilität und weniger häufigem selbstständigen Autofahren verknüpft waren. Beeinträchtigungen der Mobilität engten den Aktionsradius ein.

Das Ziel der gesundheitlichen Versorgung im Alter ist es, Unabhängigkeit und Lebensqualität der Menschen lange zu erhalten. Autofahren ist ein wichtiger Teil der Alltagsaktivitäten älterer Menschen. Deshalb sollte insbesondere hausärztlich auch regelmäßig wiederholt die Frage nach dem Autofahren gestellt werden, denn präventive Maßnahmen zum Erhalt funktionaler Gesundheit dienen auch dem Erhalt der Fahreignung älterer Menschen.

## Abstract (en)

Mobility is crucial for independent living in old age. Older people with reduced physical ability (frailty) begin to limit their personal range of activities to their immediate living environment and ultimately to their immediate home. Diseases of the musculoskeletal system as well as neurological, psychological, cognitive, sensory, and circulatory disorders can limit functional competence (ability to live independently).

In the Longitudinal Urban Cohort Ageing Study (LUCAS), from which selected results are reported in this article, participants were categorized into different functional classes (*Robust*, *postRobust*, *preFrail*, *Frail*) using the LUCAS functional index. The results show that losses in functional competence were associated with impaired mobility and reduced car driving. Impaired mobility led to restricted radius of action.

The aim of healthcare in old age is to preserve independence and quality of life as long as possible. Car driving is an important part of older peoples’ activities of daily living. Therefore, primary care physicians should address car driving regularly because preventive measures to strengthen functional health also strengthen car driving ability in older persons.

## Einleitung

Mobilität als eine der grundlegendsten alltagsrelevanten Funktionen ist auch im höheren Alter maßgeblich für die selbstständige Lebensführung. Sie kann grob als „Mittel zum Zweck“ definiert werden, Distanzen zwischen verschiedenen Orten im Raum zu überwinden. Wechselwirkungen zwischen raumstrukturellen, soziodemografischen und gesundheitsbezogenen Faktoren bestimmen Mobilitäts- und Verkehrsverhalten [1]. Folgende Bereiche sind in Betracht zu ziehen: somatische und psychische, subjektive und funktionale Gesundheit.

Die funktionale Gesundheit ist für die Altersmedizin von zentraler Bedeutung. Das biologische Alter kann bekanntlich erheblich vom kalendarischen Alter abweichen. Mit längerer Lebensdauer steigt zwar die Möglichkeit, mehrere und v. a. chronische Krankheiten aufzuweisen (Multimorbidität), aber Altern ist ein lebenslanger Prozess und nicht per se Krankheit; Organe und ihre Funktionen bzw. physiologische Regelkreise altern nicht synchron. Multimorbidität ist allerdings ein Risikofaktor für Fähigkeitsstörungen, die in der ICF (International Classification of Functioning, Disability and Health) der Weltgesundheitsorganisation (WHO) klassifiziert werden. Das komplexe Zusammenwirken von physiologischen Altersveränderungen, Krankheitsfolgen und Kontextfaktoren begründet die ausgeprägt hohe interindividuelle Variabilität im höheren Lebensalter. Das Maß vorhandener funktionaler Kompetenz (auch intrinsische Kompetenz genannt; verstanden als Gesamtheit der Kompetenzen, unabhängig zu leben) variiert entsprechend stark. Für den einzelnen Menschen ist es entscheidend wichtig, trotz altersassoziierter Veränderungen und Krankheitsfolgen selbstständig und selbstbestimmt mit Lebensfreude leben zu können [2, 3].

Eine Beeinträchtigung der Mobilität hat abhängig von Art und Schweregrad negative Auswirkungen auf die selbstständige Fortbewegung im häuslichen sowie im außerhäuslichen Bereich. Mobilitätsstörungen können so zur Einschränkung der Teilnahmefähigkeit am gesellschaftlichen Leben führen und haben ungünstige Auswirkungen auf die Lebensqualität [4]. Die gewählte räumliche Umgebung, innerhalb der Menschen mobil sind, wird auch als Aktionsraum bezeichnet [5]. Ältere Menschen, deren körperliche Leistungsfähigkeit geringer wird, beginnen ihre persönlichen „Raumbezüge“ auf das nähere Wohnumfeld und schließlich die unmittelbare Häuslichkeit zu begrenzen. Ihr individueller Aktionsraum wird enger [6]. Mögliche Beeinträchtigungen der Mobilität durch Krankheiten sind ausgesprochen vielfältig, z. B. Erkrankungen des Bewegungsapparates, neurologische, psychische, kognitive, Sinnes- oder Kreislaufstörungen, die mit Verlusten funktionaler Kompetenz einhergehen. Die systematische Untersuchung von Phänomenologie, Ursachen, Mechanismen und Auswirkungen des Verlustes von Fähigkeiten, beobachtbar als „Gebrechlichkeit“, führte zum Konzept der Frailty, dem problematischsten Aspekt der Alterung [7]. Verstanden wird darunter allgemein erhöhte Vulnerabilität mit eingeschränkter Fähigkeit des Organismus zur Beseitigung interner sowie externer Störungen – mit der Folge eines erhöhten Risikos für unerwünschte Ereignisse („adverse events“) sowie Komplikationen [7]. Die Operationalisierungen von Frailty unterscheiden sich methodisch (Phänotyp-Modell [8], kumulatives Defizit-Modell [9], umfassendes geriatrisches Assessment [10]). Mobilitätsparameter sind ein bedeutender Bestandteil der Operationalisierung. Die Literatur zu Epidemiologie von Frailty (z. B. [11]), Folgen (z. B. [9]) und Interventionen (z. B. [12]) ist mittlerweile umfangreich. Zielgerichtete körperliche Bewegung setzt u. a. auch ausreichend Muskelkraft voraus, die im Lebensverlauf abnimmt und u. U. zur Sarkopenie (altersbedingter Verlust von Muskelmasse und Muskelkraft) führt [13]. Sarkopenie ist deshalb ein wichtiger Mechanismus zur Entwicklung von Frailty.

Alltagsrelevante Mobilitätsaktivitäten älterer Menschen schließen selbstständiges Autofahren ein. Aktuelle Studien berichten, dass 30 % einer Befragungsstichprobe 80-jähriger und älterer Menschen [14] und 58 % der Patientinnen und Patienten ab 65 Jahren in einer prospektiven Beobachtungsstudie angaben, selbst Auto zu fahren [15]. Der Pkw ist auch in den höheren Altersklassen das am häufigsten benutzte Verkehrsmittel. Fast die Hälfte von 60- bis 64-Jährigen und 22 % von 75-jährigen und älteren Personen berichteten, den Pkw täglich zu nutzen [16]. Zwar waren 65-jährige und ältere Menschen im Verhältnis zu ihrem Bevölkerungsanteil 2021 unterproportional an Verkehrsunfällen mit Personenschäden beteiligt (15 %). Aber ihre Verletzungsschwere ist öfter hoch und ältere in Unfälle verwickelte Pkw-Fahrerinnen und Fahrer trugen in 68 % die Hauptschuld [17].

Die steigende Zahl älter werdender Menschen, auch chronisch Kranker, die fortgeschrittene Krankheitsstadien und Frailty erleben, erhöht die Anforderungen angemessenen Umgangs mit Fragestellungen zu Fahreignung und Fahrtauglichkeit im Alter. Zum selbstständigen Autofahren nicht mehr fähige Personen zu erkennen, ist wegen der multiplen möglichen Ursachen im höheren Lebensalter eine interdisziplinäre Aufgabe. Bestimmte Gesundheitsstörungen sind eindeutig und unabhängig vom Lebensalter nicht mit dem Autofahren vereinbar (siehe auch Beiträge von Thayssen und Püschel sowie Rieß et al. in diesem Themenheft). Schwieriger ist eine Beurteilung bei beginnender Beeinträchtigung und Kombinationen funktionaler Verluste im Rahmen der Entwicklung von Gebrechlichkeit. Die frühzeitige Erkennung beginnender Gebrechlichkeit ist jedoch bedeutsam, um Selbstständigkeit durch geeignete Maßnahmen erhalten zu können.

Dieser Beitrag soll die Bedeutung der funktionalen Gesundheit für Mobilität, Unabhängigkeit und selbstständiges Autofahren älterer Menschen unterstreichen und die Aufmerksamkeit hierfür im Zusammenhang mit Fragen der Fahreignung im Alter stärken. Im Zeitraum 2000/2001 wurden in Hamburg die Teilnehmerinnen und Teilnehmer für eine prospektive Langzeit-Kohorten-Studie, die Longitudinal Urban Cohort Ageing Study (LUCAS), zur Beobachtung individueller Alternsverläufe rekrutiert. Sie wurden bezüglich ihrer funktionalen Kompetenz den Gruppen definierter Funktionsklassen zugeordnet. Befunde aus dieser Studie zum Mobilitätsverhalten sowie auch die Merkmale selbstständig und nichtselbstständig autofahrender Teilnehmerinnen und Teilnehmer werden in diesem Beitrag mit Bezug zu den Funktionsklassen verglichen. Die Ergebnisse werden mit denen anderer Studien zum Autofahren älterer Personen diskutiert.

## Die Längsschnittstudie LUCAS

LUCAS entwickelte sich seit 2000/2001 aus der Hamburger Kohorte einer europäischen Interventionsstudie [18] zu einer noch laufenden Langzeitstudie, bei der individuelle Alternsverläufe beobachtet wurden [19–21]. Initial eingeschlossen wurden 3326 zu Hause lebende Personen ab 60 Jahren, ohne Hilfsbedürftigkeit bei den basalen Aktivitäten des täglichen Lebens (BADL), ohne Pflegestufe (gem. MDK-Einstufung), ohne demenzielle Erkrankung (Mini Mental Status ≤ 24 Pkt.) und ohne terminale Erkrankung und mit Beherrschung der deutschen Sprache. Sie wurden über ihre Hausarztpraxen rekrutiert.

Im Rahmen dieser Langzeitstudie wurde der LUCAS-Funktions-Index entwickelt [22]. Dieser zeigt keine funktionale Beeinträchtigung an und ist kein Frailty-Index, weil der Ansatz in LUCAS nicht ausschließlich von einem Defizit-Modell ausgeht. Bezogen auf veränderbare Funktionalität wird in LUCAS hingegen von einer im Verlauf des Älterwerdens variablen Balance zwischen Reserven und Risiken ausgegangen. Die Kriterien des Frailty-Phänotyps nach Fried [8] sind modifiziert enthalten. Operationalisiert ist dies in einem Selbstausfüll-Fragebogen mit Fragen zu 6 Risiken und 6 Reserven. Aus dem LUCAS-Funktions-Index können die Funktionsklassen *Robust, postRobust, preFrail *und *Frail* abgeleitet werden (siehe Infobox).

Adjustiert für Alter und Geschlecht sind diese Funktionsklassen im 8‑Jahres-Verlauf hochsignifikant prädiktiv für den Eintritt in Pflegebedürftigkeit gemäß MDK-Einstufung sowie für Überleben [22]. Damit kann die dynamische Entwicklung von Gebrechlichkeit (Frailty-Syndrom) ansatzweise beschrieben werden.

### Ergebnisse aus LUCAS zu Mobilitätsverhalten, selbstständigem Autofahren und Aktionsraum

#### Mobilitätsverhalten.

Im Rahmen der LUCAS-Befragungswelle 2011/2012 waren von insgesamt 1417 Personen (63 % Frauen) im Alter von 70,9 bis fast 102 Jahren auf Basis ihrer Beantwortung des FI-Fragebogens 544 der Funktionsklasse *Robust* (38 %) zugeordnet, 446 der Funktionsklasse *Frail* (32 %) und 427 entfielen auf die Funktionsklassen *postRobust/preFrail* zusammengenommen (30 %). Für diese 3 Gruppen zeigt die Tab. 1 deutlich unterschiedliche Häufigkeitsmuster für 4 Fortbewegungsmodalitäten und 2 beispielhafte Mobilitätsaktivitäten. Zudem sind unterschiedliche Häufigkeiten für Stürze und für die Angabe veränderter Tätigkeiten durch Sturzangst zu erkennen.

**Tab. 1:** Mobilitätsverhalten von LUCAS-Teilnehmerinnen und -Teilnehmern (*N* = 1417), Befragungswelle 2011/2012

Merkmale	Gesamt (*n* = 1417)	*Robust* (*n* = 544)	*postRobust/preFrail* (*n* = 427)	*Frail* (*n* = 446)
Alter (J.), Mittelwert (Min.–Max.)	79,0 (70,9–101,8)	76,1 (70,9–100,9)	79,4 (71,0–101,8)	82,0 (71,4–99,5)
Frauen, *n* (%)	894 (63,1)	284 (52,2)	280 (65,6)	330 (74,0)
Männer, *n* (%)	523 (36,9)	260 (47,8)	147 (34,4)	116 (26,0)
*Fortbewegungsmodalitäten, n (%)*				
Selbstständig 500 m Gehen ohne Schwierigkeit	989 (69,8)	516 (95,4)	318 (74,5)	152 (34,2)
Fahrradfahren	557 (39,3)	423 (77,8)	102 (23,9)	32 (7,2)
Nutzung ÖPNV	1105 (78,0)	470 (86,4)	339 (79,4)	296 (66,4)
Selbstständiges Autofahren	633 (44,7)	358 (65,9)	172 (40,3)	103 (23,1)
*Mobilitätsaktivitäten, n (%)*				
Selbstständig Aktivitäten außer Haus besuchen ohne Schwierigkeit	1032 (72,8)	531 (97,6)	332 (77,8)	169 (37,9)
Selbstständig Hausarztpraxis besuchen ohne Schwierigkeit	1118 (79,0)	536 (98,5)	364 (85,2)	218 (48,9)
*Stürze/Sturzangst, n (%)*				
Innerhalb der letzten 12 Monate gestürzt	414 (29,2)	86 (15,8)	112 (26,2)	216 (48,4)
Tätigkeit wegen Sturzangst verändert^a^	457 (32,2)	23 (4,2)	115 (25,2)	319 (71,5)

Hinweise: Funktionsklassen *Robust, postRobust, preFrail, Frail* ermittelt mit LUCAS-Funktions-Index [20], *ÖPNV* Öffentlicher Personennahverkehr

^a^Fragestellung: „Schränken Sie gewisse Tätigkeiten ein, weil Sie Angst haben hinzufallen?“ (Ja/Nein)

Erwartungsgemäß gaben als *Robust* klassifizierte Personen am häufigsten an, ohne Schwierigkeit zu gehen (95 %), Fahrrad zu fahren (78 %), den öffentlichen Personennahverkehr (ÖPNV) zu nutzen (86 %) oder selbstständig Auto zu fahren (66 %). Abgesehen von wenigen Personen gab diese Gruppe an, keine Schwierigkeit zu haben, selbstständig Aktivitäten außer Haus oder die hausärztliche Praxis zu besuchen, und nur etwa 4 % von ihnen gaben an, wegen Sturzangst Tätigkeiten geändert zu haben. Von den als *Frail* klassifizierten Personen gab nur ein gutes Drittel selbstständiges Gehen ohne Schwierigkeit an, Fahrradfahren 7 %, die Nutzung des ÖPNV 66 % und selbstständiges Autofahren 23 %. Die Hausarztpraxis oder Aktivitäten außer Haus selbstständig ohne Schwierigkeit zu besuchen gaben nur 38 % an. In dieser Gruppe hatte fast die Hälfte angegeben, gestürzt zu sein, und gut 70 % gaben Tätigkeitsänderungen wegen Sturzangst an. Weniger Personen mit Funktionsklasse *postRobust/preFrail* (mit zunehmenden Risiken und abnehmenden Reserven) als jene der Gruppe *Robust* gaben Gehen ohne Schwierigkeit, Fahrradfahren, selbstständiges Autofahren (40 %) und ÖPNV-Nutzung an. Nur gut 3 Viertel von ihnen gaben an, selbstständig ohne Schwierigkeit Aktivitäten außer Haus zu besuchen. Stürze nannten 26 % und ein Viertel gab an, Tätigkeiten wegen Sturzangst verändert zu haben.

#### Selbstständiges Autofahren.

Von 839 Frauen gaben 221 an, selbstständig Auto zu fahren (26 %), und 365 von den 502 Männern (73 %). In der Tab. 2 sind Merkmale selbstfahrender und nichtselbstfahrender Personen gegenübergestellt. Die 586 Selbstfahrenden verteilten sich wie folgt auf die Funktionsklassen: *Robust* 58 %, *postRobust/preFrail* 27 % und *Frail* 16 %, auf die 755 Nichtselbstfahrenden entsprechend 24 %, 33 % und 43 %. Mehr Selbstfahrende als Nichtselbstfahrende gaben ausgezeichnetes bis gutes Sehen und tendenziell auch entsprechendes Hören an. Gleichzeitig gaben aber auch 36 % aller Frauen und 29 % aller Männer eine Verschlechterung des Sehens an. Insgesamt weniger Männer als Frauen gaben ausgezeichnetes bis gutes Hören an, etwa ein Viertel aller Frauen und Männer auch eine Hörverschlechterung. Medikamentenanwendung war häufiger von den Nichtselbstfahrenden als Selbstfahrenden angegeben (Median Anzahl Medikamente 4 versus 3). Weniger als 10 % aller Teilnehmenden verneinten explizit Medikamentenanwendung. Nichtselbstfahrende (43 %) hatten deutlich häufiger als Selbstfahrende (17 %) Veränderungen bestimmter Tätigkeiten wegen Sturzangst angegeben. Dies könnte ein Hinweis sein auf vorhandene Selbstwahrnehmung, die zu Verhaltensänderungen führt, eben auch zum Verzicht auf selbstständiges Autofahren.

**Tab. 2:** Merkmale selbstfahrender und nichtselbstfahrender LUCAS-Teilnehmerinnen und -Teilnehmer, Befragungswelle 2011/2012

	Selbstfahrend (*n* = 586)		Nichtselbstfahrend (*n* = 755)	
Merkmale	Frauen (*n* = 221)	Männer (*n* = 365)	Frauen (*n* = 618)	Männer (*n* = 137)
Alter (J.), Mittelwert (Min.–Max.)	76,4 (71,0–90,7)	77,5 (71,2–92,2)	80,1 (70,9–101,8)	81,3 (71,0–100,9)
*Sehen, n (%)*				
Ausgezeichnet bis gut	154 (71,3)	272 (76,2)	352 (59,5)	72 (53,7)
Mittel bis schlecht	61 (28,2)	85 (23,8)	216 (36,5)	50 (37,3)
Sehr schlecht	1 (0,5)	0 (0,0)	24 (4,0)	12 (9,0)
Keine Angabe, *n*	5	8	26	3
*Verschlechterung Sehvermögen, n (%)*				
Keine Verschlechterung^1^	138 (67,3)	256 (73,6)	349 (62,2)	78 (64,5)
Verschlechterung^2^	67 (32,7)	92 (26,4)	212 (37,8)	43 (35,5)
Keine Angabe, *n*	16	17	57	16
*Hören, n (%)*				
Ausgezeichnet bis gut	151 (68,9)	198 (55,3)	367 (61,9)	71 (52,6)
Mittel bis schlecht	67 (30,6)	160 (44,7)	209 (35,2)	59 (43,7)
Sehr schlecht	1 (0,5)	0 (0,0)	17 (2,9)	5 (3,7)
Keine Angabe, *n*	2	7	25	2
*Verschlechterung Hören, n (%)*^*4*^				
Keine Verschlechterung	139 (73,5)	262 (76,6)	421 (74,5)	94 (75,2)
Verschlechterung	50 (26,5)	80 (23,4)	144 (25,5)	31 (24,8)
Keine Angabe, *n*	32	23	53	12
*Medikamente/Person, n (%)*				
Anzahl, Median	3	3	4	4
Keine	19 (9,5)	33 (9,4)	30 (5,4)	5 (4,0)
1–5	148 (74,4)	254 (71,9)	375 (68,2)	78 (62,4)
≥ 5 Medikamente	32 (16,1)	66 (18,7)	145 (26,4)	42 (33,6)
Keine Angabe, *n*	22	12	68	12
*Tätigkeit wegen Sturzangst verändert*^*5*^*, n (%)*				
Ja	48 (21,7)	50 (13,7)	264 (42,7)	60 (43,8)
Keine Angabe, *n*	0	1	0	0

Hinweis: Prozentangaben beziehen sich auf Teilnehmende mit Angaben

^1^Fragestellung: „Wie beurteilen Sie zurzeit Ihr Sehvermögen mit beiden Augen (ggf. mit Ihrer Brille oder Ihren Kontaktlinsen)?“

^2^Fragestellung: „Haben Sie in letzter Zeit eine Verschlechterung Ihres Sehvermögens festgestellt?“ (Ja/Nein)

^3^Fragestellung: „Wie beurteilen Sie zurzeit Ihr Hörvermögen (ggf. mit Ihrem Hörgerät)?“

^4^Fragestellung: „Haben Sie in letzter Zeit eine Verschlechterung Ihres Hörvermögens festgestellt?“ (Ja/Nein)

^5^Fragestellung: „Schränken Sie gewisse Tätigkeiten ein, weil Sie Angst haben hinzufallen?“

#### Aktionsraum und funktionale Kompetenz.

**Abb. 1:**
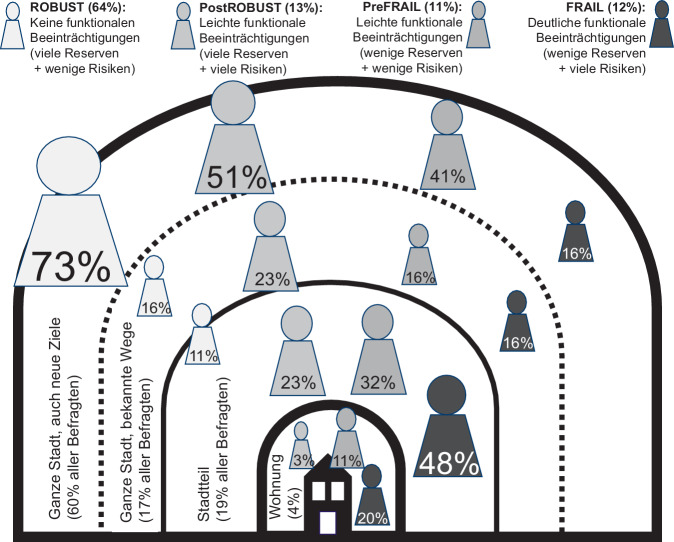
Mobilitätsaktionsräume älterer Menschen. Ergebnisse der Longitudinal Urban Cohort Ageing Study (LUCAS), Befragungswelle 2007/2008. Hinweis: Funktionsklassen *Robust*, *postRobust*, *preFrail*, *Frail* ermittelt mit LUCAS-Funktions-Index [20]. Quelle: eigene Abbildung modifiziert v. Renteln-Kruse et al. [37]

Die Abb. 1 zeigt den Zusammenhang zwischen Funktionsklasse und Reichweite im Aktionsradius. Zusätzlich zum Funktions-Index hatten 875 Teilnehmerinnen und Teilnehmer der LUCAS-Befragungswelle 2007/2008 zusätzlich Fragebögen zum Sturzrisiko [23, 24] und ihrem individuellen Aktionsraum ausgefüllt. Teilnehmende der Gruppe *Robust* zeigten größere Aktionsräume und waren überwiegend in der gesamten Stadt auch zu neuen Zielen unterwegs (73 %). Teilnehmende der Gruppe *Frail* hielten sich hingegen überwiegend im Stadtteil (48 %) oder im eigenen Zuhause auf (20 %). Die Höhe des Sturzrisikos (Anzahl von Risikofaktoren) ist eng mit der Reichweite des genutzten Aktionsradius verknüpft. Mit steigendem Sturzrisiko verkleinert sich der Aktionsradius. Verringerte körperliche Aktivität führt i. d. R. jedoch auch zum (weiteren) Verlust von Fähigkeiten. Wachsende Sturzangst kann zu Verhaltensänderungen führen und so funktionale Kompetenz verringern [4].

## Diskussion

Der wichtigste Befund ist, dass selbstfahrende LUCAS-Teilnehmerinnen und -Teilnehmer deutlich häufiger funktional fitte Personen waren, was den Bereich der Mobilität betrifft. Der Anteil der Teilnehmerinnen und Teilnehmer, die selbstständiges Autofahren angaben, verringerte sich von 66 % der Gruppe *Robust* über 40 % der Gruppe *postRobust/preFrail* auf 23 % in der Gruppe *Frail*. Vergleichbar verringerten sich die Angaben zu selbstständigem 500 m-Gehen ohne Schwierigkeit und zum Fahrradfahren. Stürze wurden am seltensten in der Gruppe *Robust* angegeben und Personen dieser Gruppe gaben auch kaum Aktivitätsänderungen wegen Sturzangst an. Während über die Hälfte der Selbstfahrenden auf *Robust-*klassifizierte Personen entfiel (58 %), verteilten sich 3 Viertel der Nichtselbstfahrenden auf die Funktionsklassen *postRobust/preFrail* und *Frail* (76 %). Die weite Altersstreuung von 71 bis über 99 Jahre in allen Funktionsklassen weist darauf hin, dass dem kalendarischen Alter keine entscheidende Bedeutung zukommt. Weiter bestätigen die Ergebnisse auch die alltagsrelevante Bedeutung von Mobilität für die Reichweite des aktiven Aktionsraums älterer Menschen.

Diese Ergebnisse passen ergänzend zu anderen Studien, die Häufigkeit und Merkmale selbstständig autofahrender älterer Menschen untersuchten.

Von 80- bis 84-jährigen Menschen aus einer Studie in Nordrhein-Westfalen gaben 59 % der Männer und 29 % der Frauen an selbst zu fahren. Bis zum Alter von 90 Jahren und älter verringerte sich der Anteil der Selbstfahrenden auf 23 % der Männer und 2 % der Frauen. Selbstfahren war verbunden mit männlichem Geschlecht, niedrigerem Alter, verheiratet und zusammenlebend zu sein, besserer Selbsteinschätzung der eigenen Gesundheit und höherem Punktwert für die instrumentellen Aktivitäten des täglichen Lebens als ein funktionales Merkmal [14].

Im Rahmen einer Studie mit Patientinnen (69 %) und Patienten im mittleren Alter von 90,3 ± 2,7 Jahren in hausärztlicher Versorgung (AgeCoDe, weitgeführt als AgeQualiDe) wurde Selbstfahren im Zusammenhang mit gesundheitsbezogener Lebensqualität untersucht. Lebensqualität wurde ermittelt mit dem Messinstrument Euro Qol EQ-5D („mobility, self care, usual activities, pain/discomfort und anxiety/depression“). Im Ergebnis gaben Selbstfahrende auch in dieser Untersuchung weniger Probleme mit ihrer Mobilität und bei der Selbstversorgung, üblichen Alltagsaktivitäten sowie geringere Ängstlichkeit an als Nichtselbstfahrende [25].

In einer französischen Studie [15] wurden selbstfahrende mit nichtselbstfahrenden Frauen und Männern verglichen bezüglich der Merkmale von *Successful Ageing* [26]. Die eng miteinander verbundenen Bestandteile dieses Konzepts sind: keine größeren Krankheiten, der Erhalt funktionaler Fähigkeiten und ein aktiver Lebensstil („active engagement with life“). In der Studie wurden physiologische, psychologische und soziale Aspekte verwendet, um Successful Ageing zu charakterisieren. Die Komponenten waren: Komorbidität (≤ 3 von 16 chronische Erkrankungen), Autonomie (basale sowie erweiterte Aktivitäten des täglichen Lebens), Kognition (Mini-Mental-Status-Test – MMST), Depressivität (Depressionsrisiko) und als soziales Merkmal „Alleinleben oder nicht“. Eingeschlossen waren Patientinnen und Patienten im Alter ≥ 65 Jahren mit chronischen Schmerzen, Diabetes mellitus Typ 2 oder Vorhofflimmern. Von den selbstfahrenden Frauen und Männern wurden 24 % mit Successful Ageing klassifiziert, von den Nichtfahrenden 7 %. Selbstfahrende erfüllten die physiologischen Komponenten in 44 %, niedrigere Komorbidität in 55 % und Autonomie in 73 %, die Nichtselbstfahrenden entsprechend in 19 %, 41 % und 36 %. Die Schlussfolgerungen aus der Studie weisen darauf hin, dass Autofahren gewissermaßen „stellvertretend“ für Successful Ageing angesehen werden könnte („driving may be considered as a proxy to successful ageing“), denn es spiegele Unabhängigkeit sowie kognitive Fähigkeiten älterer Menschen und sei Mittel, um soziale Interaktionen aufrechtzuerhalten.

Ausdruck von Successful Ageing sind u. a. auch erfolgreiche „Strategien“ älter werdender Menschen in der Auseinandersetzung mit gesundheitlichen Problemen und Fähigkeitsstörungen. Neben vorhandener Resilienz sind dies: *Selektion* (Vermeidung extremer Belastung/Überforderung), *Adaptation* oder Optimierung (gefördert durch Training und Übung) und *Kompensation* (z. B. durch Einsatz von Hilfsmitteln/Unterstützung). Auf die Verkehrsteilnahme und speziell Autofahren angewandt wären entsprechend z. B. zu nennen: Fahren bei Helligkeit und weniger Verkehr, Präferenz von Fahrten in bekannter Umgebung, Fahrverzicht (Selektion), lebenslange Fahrpraxis und defensives, langsameres Fahren kürzerer Strecken (Adaptation/Optimierung) sowie Nutzung unterstützender Technologie im Fahrzeug durch passive und aktive Assistenzsysteme (Kompensation). Eine Studie mit verkehrsmedizinisch-psychologischer Diagnostik und verschiedenen praktischen Fahrübungen kam zu dem Schluss, dass der zentrale Prädiktor für den Einsatz von Kompensationsstrategien die Fähigkeit zur Wahrnehmung subjektiver Belastungen und Einschränkungen sei [27].

In einer japanischen Befragungsstudie mit 2208 über 65-jährigen Autofahrenden, von denen 192 (9 %) angegeben hatten, im Vorjahr in einen Verkehrsunfall verwickelt gewesen zu sein, konnte ein statistischer Zusammenhang dieses Ereignisses mit dem Status „pre-Frailty“ (gemäß verwendeter Checkliste) festgestellt werden. Die Schlussfolgerung war, dass der Detektion von „pre-Frailty“ auch eine Bedeutung für die Unfallverhütung zukommt [28].

## Fazit

Den möglichst langen Erhalt von Unabhängigkeit und Lebensqualität als zentrales Ziel gesundheitlicher Versorgung im Alter [2, 3] im Blick zu behalten und zu unterstützen, zählt zu den Aufgaben insbesondere der hausärztlichen Betreuung älterer Patientinnen und Patienten. Ein wichtiger Teil alltagsrelevanter Fähigkeiten für ältere Menschen ist das selbstständige Autofahren, weshalb Hausärztinnen und -ärzte regelhaft in Abständen danach fragen sollten. Ihnen kommt eine wichtige, wenn nicht die Primärfunktion in der Ansprache des Themas „Mobilität“ einschließlich Verkehrssicherheit/Fahreignung zu [29, 30]. Präventionsmaßnahmen und Maßnahmen zur Aufrechterhaltung und/oder Verbesserung der funktionalen Kompetenz dienen auch dem Erhalt der Fahreignung älter werdender Menschen [31, 32]. Hierzu zählen unbedingt auch fachärztliche Seh- und Hörprüfungen [33–36] sowie ggf. die Einleitung weiterführender neuropsychologischer Diagnostik bei Verdacht auf beginnende kognitive Beeinträchtigungen.

### Infobox Fragestellungen zu Risiken und Reserven zur Messung des LUCAS-Funktions-Index und Ableitung von Funktionsklassen

6 Fragen zu Risiken

- Unbeabsichtigt 5 kg oder mehr an Gewicht abgenommen? (Ja)
- Art und Weise verändert, 1 km zu Fuß zu gehen? (Ja)
- Art und Weise verändert, 10 Treppenstufen zu steigen? (Ja)
- Art und Weise verändert, in ein Auto, in einen Bus/Zug einzusteigen? (Ja)
- An maximal 2 Tagen der vergangenen Woche zu Fuß unterwegs gewesen? (Ja)
- In den letzten 12 Monaten hingefallen? (Ja)

6 Fragen zu Reserven

- 500 m Gehstrecke selbstständig und ohne Schwierigkeit möglich? (Ja)
- An mindestens 3 Tagen der vergangenen Woche zu Fuß unterwegs gewesen? (Ja)
- Mindestens 1 ×/Woche mäßig anstrengender Sport? (Ja)
- Mindestens 1 ×/Woche stärker anstrengender Sport? (Ja)
- Ehrenamtliche Tätigkeit? (Ja)
- Vermeidung von Tätigkeiten wegen Sturzangst? (Nein)

Ableitung der Funktionsklassen:

- *Robust:* viele Reserven (3–6) und wenige Risiken (0–2)
- *postRobust:* viele Reserven (3–6) und viele Risiken (3–6)
- *preFrail:* wenige Risiken (0–2) und wenige Reserven (0–2) und
- *Frail:* viele Risiken (3–6) und wenige Reserven (0–2)
